# Health Literacy and Disaster Preparedness: The Role of Hazard Map Awareness among Individuals with Physical Disabilities

**DOI:** 10.31662/jmaj.2024-0347

**Published:** 2025-06-06

**Authors:** Kyo Takahashi, Kenji Takaki, Shutaro Koyama, Jun Suzurikawa

**Affiliations:** 1 Institute of Integrated Research, Institute of Science Tokyo, Tokyo, Japan; 2 Institute of Gerontology, The University of Tokyo, Tokyo, Japan; 3 Faculty of Human Ecology, Wayo Women’s University, Chiba, Japan; 4 Faculty of Human Sciences, Waseda University, Saitama, Japan; 5 Research Institute, National Rehabilitation Center for Persons with Disabilities, Saitama, Japan

**Keywords:** Health literacy, disability, disaster preparedness, hazard map, Japan

## Introduction

Everyone must take measures against natural disasters, including earthquakes, landslides, and floods, and individuals with physical disabilities are no exception ^(1)^. Understanding the risks of various natural disasters in advance and preparing appropriately based on one’s environment and health condition is critical for disaster response, evacuation, and post-disaster recovery ^(2)^. Hazard maps, which provide information on areas likely to be affected by natural disasters and evacuation places, play a key role in raising awareness about disaster preparedness and mitigating damage during such events ^(3)^. In Japan, the Flood Control Act made it mandatory for municipalities to create and disseminate hazard maps in 2005 ^(4)^. However, the extent to which individuals with physical disabilities―who often require additional support during disasters―utilize these hazard maps remains unclear.

“Health literacy is linked to literacy and entails people’s knowledge, motivation and competences to access, understand, appraise, and apply health information in order to make judgments and take decisions in everyday life concerning healthcare, disease prevention and health promotion to maintain or improve quality of life during the life course” ^(5)^. Research has identified health literacy as both a hidden risk factor for disease progression and an asset for disease prevention ^(6)^. While research on health literacy has expanded, there has been limited investigation into its role in disaster preparedness, particularly for individuals with physical disabilities ^(7)^.

Thus, this study aimed to examine the relationship between health literacy and the use of hazard maps among persons with physical disabilities. The findings are expected to contribute to improving disaster preparedness for this population.

## Materials and Methods

### Participants and data collection

The participants were recruited from an internet survey company’s database of monitors across Japan, all of whom had physical disabilities affecting the upper limbs, lower limbs, or trunk. Individuals <20 years of age or without a disability certificate were excluded. First, we estimated the required sample size to be 200, considering the number of independent variables to be entered into the logistic regression analysis and accounting for the possibility of missing data and participants meeting the exclusion criteria. Second, we confirmed the feasibility of data collection with the internet survey company. In January 2023, eligible monitors were invited via email to participate in the survey, and those who consented completed the questionnaire online. We regularly checked the status of the collected data until the target sample size was reached.

### Questionnaire

A structured questionnaire, developed by the research team, included closed questions on age, sex, living status, use of helper services, independence in activities of daily living (ADLs), health literacy, and disaster preparedness. ADL categories included mobility, urination, defecation, eating, bathing, and sleeping. Health literacy was assessed using the Communicative and Critical Health Literacy (CCHL) scale ^(8)^, a five-item scale designed to measure health literacy in the general population, with higher scores indicating greater health literacy. Disaster preparedness was assessed by asking participants if they had ever checked a hazard map for their area of residence.

### Statistical analysis

Following previous studies ^(9), (10)^, we treated health literacy as a categorical variable. We used the median total CCHL scores (high: ≥20, low: <20) as a cut-off because the data were not normally distributed (Shapiro-Wilk test p < 0.05). The concept of health literacy is more accurately grasped by looking at the relative difference between high and low scores rather than the difference between individual points. Age was treated as a continuous variable, while all other variables were treated as dichotomous. First, binary analysis (*t*-test or χ^2^ test) was conducted to explore the association between hazard map checking and other variables. Then, hierarchical logistic regression was performed to assess the impact of health literacy on disaster preparedness, adjusting for age, sex, living status, use of helper services, and ADL. All analyses were performed using SPSS v. 27.0 (IBM Corp., Armonk, NY, USA).

## Results

A total of 207 participants responded to the survey, all of whom were ≥20 years old. Of these, data from 204 participants who held an official certificate of physical disability were used (98.6%). The causes of their disabilities varied, including spinal cord injury, cerebrovascular disease, and musculoskeletal disorders.

Table 1 presents the participants’ characteristics and the results of the binary analysis regarding hazard map checking. Among the 204 respondents (113 men), 153 (75.0%) reported accessing a hazard map for their residential area. Regarding health literacy, 123 participants (60.3%) had a CCHL score at or above the median, whereas 81 (39.7%) had a CCHL score below the median. Health literacy emerged as a significant factor associated with hazard map checking. Table 2 presents the results of the hierarchical logistic regression analysis. In model 1, after adjusting for biological factors, health literacy remained a significant factor (adjusted odds ratio [AOR] 2.06, 95% confidence interval [CI]: 1.08-3.93). In model 2, which adjusted for biological and environmental factors, the AOR for health literacy was 2.12 (95% CI: 1.10-4.10). Even after further adjustment for ADLs (model 3), health literacy remained significant, with an AOR of 1.99 (95% CI: 1.01-3.92).

**Table 1.** Binary Analysis between the Participants’ Characteristics and Hazard Map Checking (N = 204).

Characteristics			Hazard map		
n (%)		Total	Checked 153 (75.0)	Not checked 51 (25.0)	p-Value
Health literacy	Low	81 (39.7)	54 (66.7)	27 (33.3)	**0.03**
	High	123 (60.3)	99 (80.5)	24 (19.5)	
Biological factors					
Age	Mean ± SD	46.9 ± 12.4	47.4 ± 12.7	45.5 ± 11.5	0.34
Sex	Male	113 (55.4)	85 (75.2)	28 (24.8)	0.94
	Female	91 (44.6)	68 (74.7)	23 (25.3)	
Environmental factors					
Living status	Alone	49 (24.0)	36 (73.5)	13 (26.5)	0.78
	Not alone	155 (76.0)	117 (75.5)	38 (24.5)	
Helper service	Using	72 (35.3)	54 (75.0)	18 (25.0)	1.00
	Not using	132 (64.7)	99 (75.0)	33 (25.0)	
ADL					
Mobility	Wheelchair	127 (62.3)	96 (75.6)	31 (24.4)	0.80
	Walking	77 (37.7)	57 (74.0)	20 (26.0)	
Urination	With support	60 (29.4)	42 (70.0)	18 (30.0)	0.29
	Independent	144 (70.6)	111 (77.1)	33 (22.9)	
Defecation	With support	66 (32.4)	46 (69.7)	20 (30.3)	0.23
	Independent	138 (67.6)	107 (77.5)	31 (22.5)	
Eating	With support	49 (24.0)	35 (71.4)	14 (28.6)	0.51
	Independent	155 (76.0)	118 (76.1)	37 (23.9)	
Bathing	With support	100 (49.0)	75 (75.0)	25 (25.0)	1.00
	Independent	104 (51.0)	78 (75.0)	26 (25.0)	
Sleeping	With support	54 (26.5)	38 (70.4)	16 (29.6)	0.36
	Independent	150 (73.5)	115 (76.7)	35 (23.3)	

ADL: activities of daily living; CCHL: Communicative and Critical Health Literacy; SD: standard deviation. *t*-test was conducted for age, and χ^2^ tests were conducted for other variables. Health literacy was divided into two groups by the median total CCHL scores (high: ≥20, low: <20). The mean ± SD of total CCHL scores was 19.4 ± 3.4 in total, 19.5 ± 3.3 in “checked” and 19.2 ± 3.7 in “not checked.” Bold indicates statistically significant differences.

**Table 2.** Hierarchical Logistic Regression Analysis Predicting Hazard Map Checking (N = 204).

	Model 1	Model 2	Model 3
	AOR (95% CI)	AOR (95% CI)	AOR (95% CI)
Health literacy	**2.06 (1.08-3.93)**	**2.12 (1.10-4.10)**	**1.99 (1.01-3.92)**
Biological factors			
Age	1.01 (0.99-1.04)	1.01 (0.99-1.04)	1.01 (0.98-1.04)
Sex	0.96 (0.50-1.84)	0.95 (0.49-1.84)	0.99 (0.50-1.94)
Environmental factors			
Living status		1.02 (0.47-2.23)	0.95 (0.43-2.12)
Helper service		0.86 (0.42-1.77)	0.86 (0.35-2.12)
ADL			
Mobility			0.88 (0.40-1.98)
Urination			1.00 (0.26-3.93)
Defecation			0.62 (0.15-2.52)
Eating			1.02 (0.33-3.19)
Bathing			1.52 (0.55-4.22)
Sleeping			0.79 (0.24-2.58)

ADL: activities of daily living; AOR: adjusted odds ratio; CI: confidence interval. Model 1: health literacy, and biological factors (age, sex). Model 2: health literacy, biological factors, and environmental factors (living status, helper service). Model 3: health literacy, biological factors, environmental factors, and ADL (mobility, urination, defecation, eating, bathing, and sleeping). Health literacy was entered as a dichotomous variable, divided by the median. The AOR (95% CI) was 1.15 (0.73-1.82) in model 1, 1.15 (0.72-1.84) in model 2, and 1.01 (0.99-1.04) in model 3 when health literacy was entered as a continuous variable of the original score. Bold indicates statistically significant differences.

## Discussion

This study found that health literacy was significantly associated with hazard map checking, even after adjusting for biological, environmental, and ADL factors. These findings highlight the crucial role of health literacy in enhancing disaster preparedness among individuals with physical disabilities, regardless of their background. Previous studies have emphasized the importance of community-related factors, such as neighborhood associations, in supporting disaster preparedness for this population ^(11), (12)^. Traditionally, people with physical disabilities have been viewed as vulnerable and in need of protection, with a focus on reinforcing external support. While this approach remains essential, it is equally important to empower individuals by strengthening their own capabilities ^(13)^. This study contributes new insights, demonstrating that capabilities such as health literacy are important for disaster preparedness.

Health literacy is especially important for people with physical disabilities in checking hazard maps due to the strong link between health and disaster preparedness in Japan. Individuals with high health literacy tend to proactively seek information beneficial to their health ^(5), (6)^. In a disaster-prone country like Japan ^(14)^, it is logical that people with higher health literacy would routinely access information, such as hazard maps, to safeguard their well-being during disasters. For those with physical disabilities―who may require equipment or assistance to maintain their health―planning for health-related needs during disasters is crucial ^(15)^. Another factor to consider is the accessibility of hazard maps. Many hazard maps, created by local governments, contain extensive information, which can be overwhelming. Individuals with physical disabilities who are indifferent to their own health or overly confident in their preparedness may not see the value in investing time and effort into reviewing these maps.

This study has some limitations. First, its cross-sectional design limits the ability to determine causal mechanisms. Longitudinal studies are needed to verify the causal relationship between health literacy and disaster preparedness. Second, selection bias cannot be ruled out because participants were recruited from an internet survey company’s database of monitors. This makes it difficult to generalize the results of this study to many people with physical disabilities, including those without access to the internet. Third, the amount of information on disaster preparedness and external factors was limited. By using questions that take into account the level of understanding of hazard maps, their usage, and regional characteristics, more detailed quantification and comprehensive analyses will be possible. Despite these limitations, this is the first study, to our knowledge, to highlight the importance of health literacy in disaster preparedness for individuals with physical disabilities. The findings have broadened the scope of health literacy research to disaster preparedness for persons with disabilities. Furthermore, they are expected to serve as evidence to promote disaster preparedness education for people with disabilities and strengthen the disaster resilience of individuals, their supporters, and society.

To promote disaster preparedness among people with physical disabilities, it is important to empower them and improve their access to information ^(13), (16)^. Initiatives aimed at enhancing health literacy could equip individuals with the skills to protect their health both during normal conditions and in the event of disasters. Additionally, efforts should be made to ensure that hazard maps are easy to access and understand, regardless of an individual’s health literacy level.

## Article Information

### Conflicts of Interest

None

### Sources of Funding

This work was supported by the Japan Agency for Medical Research and Development under Grant Number 24dk0310120h0403.

### Acknowledgement

We thank everyone at the Department of Public Health, Dokkyo Medical University, for their valuable comments.

### Author Contributions

Kyo Takahashi was involved in the study concept, design, and participant recruitment. All authors were involved in the data analysis, interpretation, and manuscript preparation.

### Approval by Institutional Review Board (IRB)

The study design was approved by the Ethics Committee of Dokkyo Medical University (approval number: 2022-013).

### Data Availability

The datasets generated and analyzed during the current study are not publicly available but are available from the corresponding author on reasonable request.
